# Using 2D and 3D pluripotent stem cell models to study neurotropic viruses

**DOI:** 10.3389/fviro.2022.869657

**Published:** 2022-07-29

**Authors:** Emma LaNoce, Jeriel Dumeng-Rodriguez, Kimberly M. Christian

**Affiliations:** 1 Mahoney Institute for Neurosciences, Department of Neuroscience, Perelman School of Medicine, University of Pennsylvania, Philadelphia, PA, United States; 2 Developmental, Stem Cell and Regenerative Biology Program, Cell and Molecular Biology Graduate Group, Perelman School of Medicine, University of Pennsylvania, Philadelphia, PA, United States

**Keywords:** iPSCs, organoids, neurotropic viruses, 3D culture, 2D culture, brain development, ESCs

## Abstract

Understanding the impact of viral pathogens on the human central nervous system (CNS) has been challenging due to the lack of viable human CNS models for controlled experiments to determine the causal factors underlying pathogenesis. Human embryonic stem cells (ESCs) and, more recently, cellular reprogramming of adult somatic cells to generate human induced pluripotent stem cells (iPSCs) provide opportunities for directed differentiation to neural cells that can be used to evaluate the impact of known and emerging viruses on neural cell types. Pluripotent stem cells (PSCs) can be induced to neural lineages in either two- (2D) or three-dimensional (3D) cultures, each bearing distinct advantages and limitations for modeling viral pathogenesis and evaluating effective therapeutics. Here we review the current state of technology in stem cell-based modeling of the CNS and how these models can be used to determine viral tropism and identify cellular phenotypes to investigate virus-host interactions and facilitate drug screening. We focus on several viruses (e.g., human immunodeficiency virus (HIV), herpes simplex virus (HSV), Zika virus (ZIKV), human cytomegalovirus (HCMV), SARS-CoV-2, West Nile virus (WNV)) to illustrate key advantages, as well as challenges, of PSC-based models. We also discuss how human PSC-based models can be used to evaluate the safety and efficacy of therapeutic drugs by generating data that are complementary to existing preclinical models. Ultimately, these efforts could facilitate the movement towards personalized medicine and provide patients and physicians with an additional source of information to consider when evaluating available treatment strategies.

## Introduction

Viral infections that impact the central nervous system (CNS) can lead to chronic injury or cognitive impairments that have a significant impact on quality of life. Due to the potential severity of neurological sequelae following infection, there is a need to determine the neurotropism of viruses and understand the underlying pathology. However, this has been challenging due to the inaccessibility of viable CNS tissue from patients, the difficulty in disentangling direct and indirect effects of infection from analyses of postmortem tissue, as well as the complexity of virus-host interactions and variability of immune responses. Identifying neural targets of viral infections and the virus-mediated causes of neurological symptoms is critical to the development of effective therapeutic or preventative strategies but remains a significant challenge within neurovirology (1).

Much of the evidence for neurotropism in humans arises from examinations of postmortem tissue of an infected individual or epidemiological studies that suggest neurological effects. Both approaches provide invaluable data and are generally the first indication that viral infections can impact the CNS. However, these studies do not allow for direct experimentation to uncover the causal mechanisms of pathology. Animal studies do allow for controlled investigations of viral infections in a physiologically intact model, but can be limited due to species-specific differences in virus-host interactions or susceptibility. Cell culture is one of the most broadly used experimental approaches in biomedical research, including virology, and is highly amenable to controlled investigations. Cell lines such as Vero, HeLa, HEK293, or SH-SY5Y have led to critical insight into the mechanisms of virus-host interactions and pathogenesis. Each of these lines has been well established in the field and offers advantages in terms of consistency, scalability, and ease of access. However, most are from non-neural lineages or derived from tumor tissue and have distinct properties that may not recapitulate cellular virus-host interactions in developing or mature human CNS cell types. Functional assays relevant to understanding the pathogenesis of neurological and cognitive symptoms may also be limited in these cell lines.

Human embryonic stem cells (ESCs) were first described in 1998 and were generated from the totipotent cells obtained from the inner cell mass of embryos at the blastocyst stage (2). Human ESCs demonstrated several defining characteristics first established in mouse ESCs, including a proliferative capacity in the undifferentiated state and the potential to generate cell types representative of all three embryonic germ layers. These properties allow for a renewable source of human cells of nearly any cell type in the body, including neural cells derived from an ectodermal lineage. Several ESC lines have been established and well-characterized but are limited in number due to restrictions on the generation of additional lines. The development of technology to reprogram human adult somatic cells into a pluripotent state reminiscent of ESCs demarcated a radical shift in the field of medical research and has opened the door for personalized medicine (3). Human induced pluripotent stem cells (iPSCs) offer several distinct advantages over ESCs, including the ability to generate new lines from consenting individuals that retain the genetic information of the donor. Given that the number of ESC lines available for research is limited, the ability to interrogate virus-host interactions in a wide variety of genetic backgrounds in iPSCs could facilitate a better understanding of genetic risk factors that can lead to more severe outcomes after viral challenges. Similar to ESC lines, iPSCs can be differentiated into almost any cell type, including neurons, glia, and immune cells (4). As such, iPSCs are an invaluable resource that can provide an unlimited source of cloned human neural cell types from individual donors, representing more genetic diversity than in existing ESC lines. However, this also suggests an even greater need to conduct studies using more than one donor line to account for the variability due to differences in reprogramming and/or genetic background.

As with any model system, there are limitations to PSC-based studies but there is also great potential to complement existing animal and cell culture models for further discovery and to identify new treatments for known and emerging neurotropic viruses (5–7). In this review, we discuss a few of the recent studies that illustrate the advantages of 2D and 3D human cell cultures that have advanced our understanding of the impact of viruses on the CNS using a repertoire of PSC-derived neural cell types and culturing techniques (Table 1). Although iPSC studies have become more popular in recent years and comprise the majority of studies we cite, ESCs are still widely used and may be subject to less genomic instability than iPSCs. We selected studies to represent a range of neurotropic viruses that also illustrate key features and recent advances in cell culture technology. Unfortunately, we were unable to cite all relevant PSC studies of neurotropic viruses due to space limitations.

## Two-dimensional cultures

Differentiating ESCs and iPSCs into an enriched monolayer culture of targeted cell populations enables controlled, rigorous, and focused experimentation. Two-dimensional (2D) models are particularly beneficial for investigations focusing on specific human cell types, advanced maturational states, defined cellular interactions, and higher-throughput phenotyping. Importantly, monolayer culturing of cells allows for consistent exposure to the factors in the media (e.g., oxygen, growth factors and nutrients, infectious agents, drug concentrations) and temporal control of proliferation to isolate various dynamic states. The ability to differentiate iPSCs into highly enriched populations of specific human neural cell types is a defining characteristic and key advantage of this *in vitro* model, especially when investigating known targets of a particular neurotropic virus.

### Cellular differentiation, enrichment, and cell-type identity

Following the demonstration that iPSCs could be generated from adult somatic cells in 2006 (94), many protocols have been developed to program these cells into early neural stem cells (NSCs) and neural progenitor cells (NPCs), as well as mature cell types representative of the CNS, including neurons (95–98), astrocytes (99, 100), microglia (101, 102), and oligodendrocytes (103, 104). Differentiation of cell types typically relies on varying the concentration and duration of exposure to patterning factors such as WNT, FGF8, TGF-β, and SHH that are critical during embryonic development (105–107). Though the efficiency of such methodologies can vary (108, 109), some protocols demonstrate the potential to generate robust, nearly 100% homogeneous populations of neural cells (99, 110). Variability can be further reduced using purification tools such as fluorescence- or magnetic-activated cell sorting to select for cell surface markers of a target population (111, 112).

It is important to note that how cell “type” is defined in a study also contributes to the degree of differentiation robustness and purity of the enriched culture (113). Seemingly homogeneous populations based on neurotransmitter expression may be comprised of many subtypes, such as highly heterogeneous GABAergic neurons or glutamatergic neurons of distinct cortical layers (114, 115). Due to the complexity of the CNS and the different ways of defining cell types (e.g., morphological, electrophysiological, molecular), the number of neural subtypes that exist in the brain has yet to be fully delineated (116). However, recent advances in single-cell sequencing technologies have revealed extensive regional and cell-type specificity, as well as species-specific differences, at a level of granularity that had not been previously appreciated (117).

### Cell type-specific viral responses

Maintaining monocultures of different cell types in parallel provides opportunities for comparative analyses of responses to viral exposure. One such example of cell type-specific susceptibility was shown in a study that tested reactivity of different iPSC-derived CNS cell types to investigate the pathogenesis of childhood herpes simplex virus 1 (HSV-1; herpesvirus) encephalitis (HSE) (23). Although HSV-1 is a widespread virus that infects a large percentage of young adults worldwide, HSE is a rare but life-threatening disorder that can result from innate genetic mutations of *TLR3* or *UNC-93B*, which play a key role in the immune response (118–120). Mutations affecting these proteins confer a selective deficiency in immunity to HSV-1, leading to HSE pathogenesis in affected patients. After reprogramming fibroblasts from donors with UNC-93B or TLR3 deficiencies, iPSCs were differentiated into NPCs, cortical neurons, astrocytes, and oligodendrocytes (23). Among all cell types, the researchers found that neurons and oligodendrocytes can provide strong anti-HSV-1 immunity *via* an intact TLR3 pathway, but these cells are highly vulnerable to HSV-1 infection if they are deficient in the TLR-specific UNC-93B membrane protein. In contrast, UNC-93B-deficient NSCs and astrocytes were not more susceptible to infection (23). These data extend findings from studies showing the importance of intact TLR3 signaling and UNC-93B function in other cell types (e.g., fibroblasts, T cells) for immunity to HSV-1, as well as the prevalence of TLR3-associated genetic variants that may contribute to HSE (120, 121). Subsequent studies have identified additional genetic modulators of innate immunity for HSV-1, specifically in cortical neurons derived from iPSCs (24). Consistent with findings from postmortem tissue, iPSC-derived trigeminal ganglion neurons both with and without TLR3 pathway mutations are highly susceptible to HSV-1 infection, demonstrating a lack of innate immunity in these neurons that are thought to be one source of HSV-1 latency and reactivation (25). HSV-1 latency and treatment has been profiled in human ESC-derived neurons as well, demonstrating high infectivity rates with a wild-type HSV-1 and the establishment of a latent state of non-productive infection when coupled with antiviral drugs (26). Together, these studies highlight the value of generating iPSC lines with patient-specific mutations for functional investigations, as well as the capacity for directed differentiation of PSCs to reveal cell type-specific tropism and innate immune responses.

### Maturation of neural cell types and developmental stage-dependent viral effects

**FIGURE 1:**
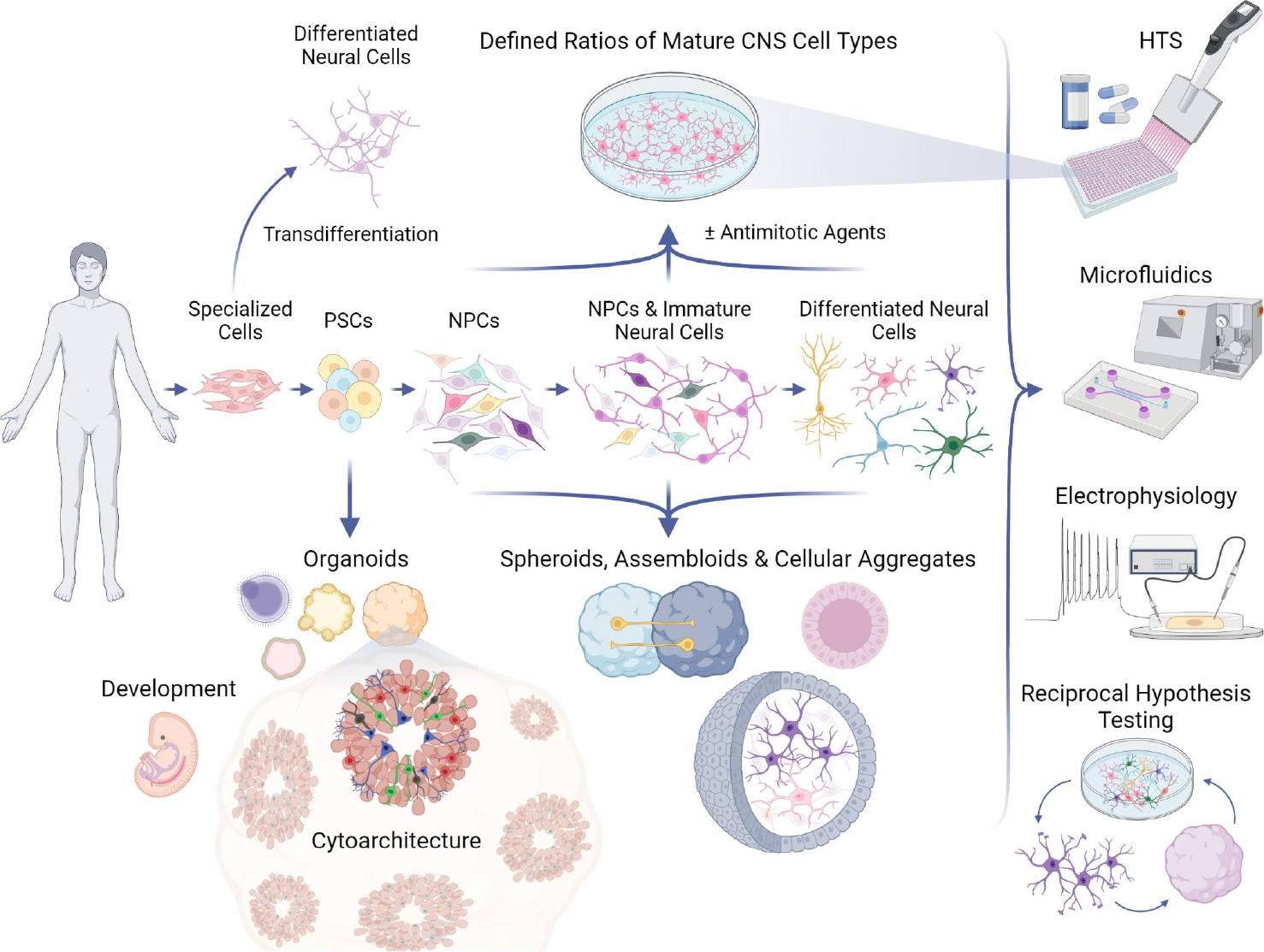
Applications of PSC models to study neurotropic viruses. Human pluripotent stem cells (PSCs) can be guided to differentiate into 2D (top) or 3D (bottom) *in vitro* neural networks, which can be used in a variety of assays, including high-throughput screenings (HTS) and electrophysiological recordings (right). PSCs can develop into various complex organoid models which can recapitulate aspects of human neural development and neuroanatomy, or 3D models of desired cell types which strive to mimic *in vivo* cell behavior and physiology. PSCs can follow differentiation protocols to become monolayers of neural progenitor cells (NPCs), immature neural cells, and ultimately any central nervous system (CNS) cell type, such as microglia, astrocytes, oligodendrocytes, and post-mitotic neurons. Antimitotic agents can be used to synchronize maturation and generate more homogeneous 2D cultures for controlled investigations that can be further elaborated in 3D models, refined again in 2D formats, and so on by way of reciprocal hypothesis testing. Specialized human cell types (e.g., fibroblasts, blood) can be transdifferentiated directly into neural cells without transitioning through a pluripotent state, providing another platform for CNS experimentation.

Another key advantage of culturing iPSC-derived cells in 2D is the capacity to generate and manipulate large populations of cells from neural lineages of a defined cellular age (95, 122–125) (Figure 1). Synchronization of differentiation can generate largely homogeneous cultures of cells in the same maturational state. Depending on the cell type, *in vitro* maturity may be attained over several weeks and can be facilitated by specialized reagents (e.g., BrainPhys medium) to accelerate synapse formation and the emergence of electrophysiological activity that may better reflect properties of postmitotic neurons *in vivo* (126, 127). Mitotic inhibitors such as cytosine arabinoside (AraC) (128) and uridine/fluorodeoxyuridine (U/FdU) (129) can synchronize maturation by eliminating NPCs from the network, especially when the inhibitors are infused intermittently to account for quiescent NPCs working to repopulate depleted progeny (128). Although these methods allow for some degree of control over the initiation of differentiation, there are still many outstanding questions as to how to define cellular maturity and the most salient features may depend on the experimental question. Typically, the state of maturation is determined through immunohistochemical analyses of protein expression or morphology, gene expression *via* RNA-sequencing (130), or activity levels *via* electrophysiological properties and calcium transients (131). Single-cell sequencing and electrophysiological assays have also shown that co-culturing NPCs and neurons with astrocytes can enhance and accelerate the expression of transcriptional signatures associated with neuronal maturation (132).

The traditional method for differentiation of iPSCs into neural cell types involves dual-SMAD inhibition that is meant to recapitulate the full neurogenic process. Although astrocytes can be generated through some of the same protocols that produce cortical glutamatergic neurons, dedicated astrocyte differentiation protocols are more efficient. However, these protocols are typically very time-intensive, with some protocols requiring more than 3 months to acquire markers of cell-type specificity and maturation. Protocols to accelerate this process have been developed for both astrocytes (133) and neurons (97), the most popular of which relies on the inducible overexpression of neurogenin 2 (NGN2), which can produce functional neurons in ~14 days (134). In a direct comparison of neurons derived from a traditional or accelerated protocol, a recent study showed that more mature electrophysiological properties could be detected in neurons from the dual-SMAD differentiation protocol than the NGN2 overexpression protocol and that terminally differentiated neurons continued to mature up to 6 months in culture (135). There are advantages and disadvantages to both types of protocols in terms of the relative length of culture time (~14 days *vs*. several weeks to months), the degree to which the developmental stages of neuronal maturation are recapitulated, and the ability to rapidly scale large populations for higher-throughput screening.

For viruses that may affect the adult CNS, there is a need to investigate postmitotic neural cell types (e.g., mature neurons, astrocytes) that better model the mature brain. Previous studies indicated that West Nile virus (WNV; flavivirus), rabies virus (RABV; rhabdovirus), varicella-zoster virus (VZV; herpesvirus), and Usutu virus (USUV; flavivirus) may target these cells both *in vivo* and *in vitro* (8, 41, 59, 136–139). Comprehensive understanding of the mechanisms underlying infection of WNV and VZV in human neurons, however, remains elusive. One of the earliest studies using differentiated neurons combined an *in vivo* model and a stem cell-based approach to study the effect of WNV, a mosquito-transmitted virus that can cause neurological consequences such as encephalitis or meningitis in humans (138). In the *in vivo* mouse model, WNV infection disseminated throughout the CNS within 4 to 6 days and correlated with the death of motor neurons in the spinal cord and symptoms of paralysis. Following the *in vivo* study, the group next examined the mechanism associated with neuronal injury using mouse ESC-derived neurons, and found that WNV infection caused apoptosis of neurons within 48 hours of infection in the absence of activated lymphocytes or microglia (138). This study provided *in vivo* data in a mouse model that was consistent with symptoms observed in WNV patients (140), and evidence of a direct effect of WNV on neurons.

Using human stem cell models at various stages of differentiation (e.g. iPSCs, NSCs, neurons), a recent study (8) compared several neurotropic viruses for cell type specificity and found that WNV most efficiently infected both neurons and NSCs compared to Zika virus (ZIKV; flavivirus) and dengue virus (DENV; flavivirus), which have shown preferential tropism for NPCs and blood-brain barrier (BBB) cells, respectively (141–144). This comparative study represents an effective approach to evaluate relative infectivity of different viruses in neural cell types and suggests that WNV replicates more rapidly in neurons and induces the highest rates of apoptosis in this population among the three related flaviviruses of interest. Though informative, more iPSC-based research could help to delineate the chief target of WNV tropism, as well as that of DENV, which has only recently been investigated for its neurotropic properties in human PSC models.

A similar study applied a 2D maturation approach to establish a model for examining VZV, a highly neurotropic, human-specific herpesvirus which can lead to varicella (chickenpox), herpes zoster (shingles), and other neurological and ocular disorders in infected individuals (59). Neuronal aggregates, or neurospheres, were produced from primary human fetal brain NSCs and dissociated to develop a largely neuronal population of 90% neurons and 5% astrocytes in a 2D system. VZV infection experiments revealed only 5–10% of neurons, and no astrocytes, consistently contained viral proteins up to 3 weeks after infection, suggesting no significant cell-cell transmission. Compared to primary fetal lung fibroblast cultures, which led to a cytopathic effect within 5–7 days post-infection, VZV did not promote a cytopathic effect or death in cultured neurons. Built upon earlier investigations of primary neuron culture and postmortem tissue models (60, 145, 146), this NSC model and other ESC-based approaches (61) helped to pave the way for more in-depth examination of VZV-neural cell interactions in humans. A study of human dorsal root ganglion xenografts in mice later found that VZV infection may persist in neurons for at least 8 weeks (147) and retain resistance to apoptosis, increasing the likelihood of latency and reactivation effects among infected cells. Others have since extended this dorsal root ganglia model to focus on iPSC-derived sensory neurons, which seem to be one of the few reservoirs of latent VZV (27). These studies demonstrate how 2D investigations of mature neuronal populations can provide foundational information on different neurotropic viruses with various degrees of aggression, informing future analyses of mature cell behavior when exposed to these and other infectious agents in a physiologically intact environment. Using these tools, researchers can further analyze how PSC-derived cells at various stages of development respond to environmental stimuli and perturbagens such as viruses or drugs, and recognize any potential effects that different cell types may exert throughout the CNS.

### Cell-cell interactions and non-cell autonomous effects of viral infections

Of particular relevance to understanding neurological consequences of viral infections is distinguishing between direct and indirect effects. Many viruses exert “bystander” non-cell autonomous effects on surrounding cells, resulting in cell death or disturbance of uninfected cells (148–151). Many studies investigating *in vitro* bystander effects of CNS cells involved in the immune response, i.e., microglia and astrocytes, utilize various models such as immortalized human cell lines, rodent models, or monocyte-derived cultures. Although these studies provide valuable information regarding basic mechanisms and processes involved in cellular interactions, patient-derived iPSCs allow for investigation of the most relevant human cell types and could provide further opportunities to investigate individual variability in susceptibility to pathology.

A striking example of the potential for non-cell autonomous effects is the impact of human immunodeficiency virus (HIV; retrovirus) on the CNS. Up to 50% of people living with HIV experience some degree of cognitive impairment even under viral suppression *via* a therapeutic regimen of antiretroviral drugs (ARVs), but the underlying cause is unclear (152). It is well known that HIV does not infect neurons directly, but does infect microglia, the resident immune cells of the CNS (16, 153, 154). Some studies have suggested astrocytes are vulnerable to infection of HIV (155, 156), while others maintain that astrocytes engulf HIV particles but are not subject to direct infection (157). Astrocytes may serve as reservoirs or conduits for persistent effects of HIV protein expression or DNA integration, potentially facilitating viral transmission among cells (e.g., macrophages, CD4^+^ T cells) *via* cell-cell contact as revealed through *in vitro* primary astrocyte cultures and patient samples (158). Further complicating the interpretation of the neural consequences of HIV infection are reports that some ARVs may be neurotoxic, potentially contributing to the persistence of HIV-associated neurocognitive disorders (HAND) (159, 160). For these reasons, it is critical to decipher the cell type-specific impact of HIV infection, as well as ARVs, in mixed cultures of all relevant cell types.

iPSC-derived co-cultures of neurons, microglia, and/or astrocytes have been established to model cell autonomous and non-cell autonomous effects of HIV infection and ARV exposure (17, 18). In a tri-culture model of all three cell types, HIV infection of microglia led to increased production of proinflammatory cytokines (e.g., IL-1β, IL-1α, TNF-α) (17), similar to what has been observed in postmortem tissue from HIV encephalitis cases that showed elevated IL-1β in infected microglia (161, 162). Although microglia showed the most robust activation of inflammatory pathways, as would be expected from direct HIV infection, elevated levels of cytokines were also observed in neurons and astrocytes suggesting a bystander effect on neighboring cells (17).

Microglia have been implicated as targets of infection from other neurotropic viruses such as Japanese encephalitis virus (JEV; flavivirus), Chandipura virus (CHPV; rhabdovirus), and some coronaviruses (23, 150, 151, 163–165). However, the CNS targets of many viruses such as JEV, a mosquito-transmitted virus that causes inflammatory disease with a 25–30% mortality rate and 50% likelihood of life-threatening neurological complications (163, 166), are not fully known. JEV appears to target developing neurons and glial cells in rodent and human ESC models (39, 167), and the viral antigen was detected in several human brain regions including the thalamus, brainstem, and hippocampus, as revealed through patient postmortem tissue collection (168). One study generated a co-culture of JEV-infected human monocyte-derived microglia with susceptible hamster fibroblasts (163). Viral transmission from infected microglia to target cells was found to be extremely sensitive to interactions within the CX_3_CR1-CX_3_CL1 axis, which is also a main regulator of chemotaxis and communication between microglia (CX_3_CR1) and neurons (CX_3_CL1) (169). The findings suggest that cell contact-mediated transmission may contribute to neuronal infection at early stages of infection, and that the CX_3_CR1-CX_3_CL1 axis may be a prime target for therapeutics in infected individuals (163). Studies such as these support the application of 2D mono- and co-cultures as opportune models for discovering potential candidates for viral remediation agents.

### High-throughput screening

The efficiency of 2D ESC and iPSC culture protocols provide a platform for high-throughput screening (HTS) formats (e.g., in 96-, 384-well plates) in which large numbers of neural cell types can be produced for molecular manipulation, phenotypic analysis, and/or drug screens (170–172). Directed differentiation of PSCs may be more suitable for HTS than immortalized cell lines, which tend to yield lower predictive measurements of toxicity, due in part to limitations in cell type-specific differentiation and function (171). The viability of developing iPSC models with a variety of genetic backgrounds makes this model particularly suitable for toxicology research and drug screening, especially when cells are obtained from patients who exhibit severe symptoms following viral infections or rare side-effects from medication (173). It would also be beneficial to standardize a platform to investigate genetic diversity in response to viral challenges and during drug development to increase the likelihood of identifying phenotypes or drug responses that are likely to reflect the majority of the population. Phenotypic assays such as high-content cell imaging provide opportunities for studying biological processes impacted by viruses, including autophagy, which can be induced by RABV and HSV-1 (174–176), mitochondrial function, which may be disrupted following ZIKV and SARS-CoV-2 infection (coronavirus) (63, 177, 178), as well as morphological analyses of neuronal development. Multi-electrode arrays can also provide functional readouts of neuronal activity in a medium-throughput format. Once a robust phenotype is identified, drug screens can be performed either in an unbiased manner using large libraries of bioactive compounds or in a hypothesis-driven approach to screen compounds known to affect specific cellular pathways based on predicted mechanisms.

Based upon previous findings suggesting ZIKV infection of NPCs results in increased caspase-3 activation and cell death (64, 74), a recent study developed a high-throughput compound-screening approach using 384- and 1,536-well plate assays and libraries comprised of over 6,000 compounds to assess efficacy in ZIKV treatment (64). The study identified two classes of compounds with antiviral and neuroprotective capabilities in iPSC-derived cultures. The pan-caspase inhibitor emricasan was found to be the most protective compound against cell death in NPCs, though it does not suppress ZIKV infection. Niclosamide, a Food and Drug Administration (FDA)-approved antiparasitic drug, and PHA-690509, a cyclin-dependent kinase inhibitor, were classified as the most effective compounds, inhibiting replication of all three tested strains of ZIKV. These results also indicate a potential benefit of combining both neuroprotective and antiviral compounds in ZIKV remediation, which was particularly effective in preserving astrocyte viability after infection.

Drug screening is also beneficial when there emerges a need to discover new therapies to replace existing, potentially ineffective strategies. In response to reports of drug resistance and neurotoxic effects of the drug acyclovir, one study compared a suite of 73 anti-herpetic drugs against HSV-1 infection in iPSC-derived neuronal lineages at different stages of development (e.g., stem cells, NPCs, neurons) and Vero cell cultures using high-content image analysis (28). The screening identified new compounds with anti-HSV-1 properties in neuronal cells such as the quinazolinone derivative CB-3–176 and demonstrated that a larger number of the drugs tested showed more inhibitory activity in neurons than in NPCs, with Vero cells expressing the lowest rates of inhibition. This study illustrates the advantages of using PSC-based models for CNS-specific drug screening, as only five of the 19 compounds that exhibited significant antiviral activity in the iPSC-derived neurons were effective in the Vero model system. For viruses like HSV-1, which express tropism for immature neuronal cells (36), examining drug effects on not only mature primary neurons but also iPSCs in earlier stages of differentiation remains essential for comprehensive drug discovery and the understanding of virus-mediated pathology in the CNS of adults and during fetal development.

A more recent development is the emerging technology associated with CRISPR-based gene editing strategies to identify causal mechanisms of viral replication and virus-mediated pathology. Targeted editing of viruses *via* CRISPR/Cas9 may provide a new therapeutic approach to eliminate viruses in various states of latency within the CNS (179). This approach can also be used to identify cellular components within the host that permit viral infections (180, 181). This is an exciting avenue and the ability to combine targeted gene editing with human neural cell types holds the promise of being able to accelerate our understanding of virus-host interactions and facilitate rational drug design.

### Limitations of 2D cultures and emerging technologies

PSC models are an invaluable resource for human-specific disease research, especially for the CNS, as it can be difficult to investigate causal mechanisms from analyses of postmortem brain samples. However, there are several caveats that may constrain some applications of 2D models. Although monolayer iPSC cultures have immense scalability, this results in a simplified, less complex model than 3D *in vitro* models and *in vivo* systems. As such, 2D models inherently express limitations in recapitulating natural physiological conditions. Cells may interact more with the culture plate substrate rather than with other cells in the network, therefore missing a landmark characteristic of *in vivo* cytoarchitecture: the extracellular matrix (ECM). A lack of ECM can alter dynamics such as nutrient and molecular gradients, polarity, migration, proliferation, morphology, and communication, which may ultimately affect cellular function and behavior (182–185). Low rates of reproducibility and efficiency for some existing differentiation protocols may also hinder cell-type specific analyses and integration of data across different research groups. Several adaptations have emerged to overcome potential challenges of 2D models and develop more physiologically relevant networks and substrates, such as nanowire arrays (186, 187), sandwich cultures (188), micropatterning of cell-adhesive islands (189), modulated substrates (190), and microfluidic devices (191). Few, however, have been applied to study iPSC-based models of neurotropic viruses. One strategy to capitalize on the intact physiological system of *in vivo* animal models to investigate viral-mediated responses in human cells is to perform xenografts of PSC-derived cells. Human PSC-derived hematopoietic progenitors and microglia have been transplanted to the rodent brain and upon engraftment, these cells can acquire molecular properties that are more representative of *in vivo* populations than is typically observed *in vitro* (192–194). Similar strategies have been used to “humanize” animals that are otherwise resistant to infection of some viruses, such as HIV (195). Transplantation of human progenitors early in development can also facilitate investigations of viral pathogenesis in the developing brain. Another rapidly evolving area of research in response to 2D constraints is the generation of organoids or assembloids from human PSC-derived cells to develop an *in vitro* model that includes multiple cell types and captures some of the structural features of particular brain regions.

## Three-dimensional cultures

Three-dimensional (3D) cell culture approaches are a more recent development in the PSC field and there is much excitement about the potential of this model to investigate emergent properties of highly organized neural structures, as well as the dynamic features of human brain development. Cerebral organoids can be derived from human iPSCs or ESCs and can recapitulate many features of cell migration, neural cytoarchitecture, and the formation of neural circuits. The cellular heterogeneity that arises from self-organizing populations of NSCs facilitates the study of neurotropism of viruses when the target cell type is unknown.

### 3D differentiation and brain-region specificity

Multiple protocols have been developed to generate organoids that model various regions of the CNS, which differ mainly in the patterning factors used and duration of directed differentiation. The earliest published protocols relied largely on intrinsic differentiation signaling that ultimately results in stochastic organoids that can resemble multiple regions of the brain (196, 197). More recently, guided differentiation protocols have been optimized that rely on the addition of specific patterning factors to generate organoids that resemble specific brain regions, such as the thalamus (198), hypothalamus (199), cerebellum (200), midbrain (201), brainstem (202), hindbrain and choroid plexus (203), cerebral cortex (75), hippocampus (204), striatum (205), and spinal cord (206), among others. Similar to directed differentiation in 2D cultures, patterning for organoids relies on establishing the appropriate concentrations of various morphogens in the cell culture media that mimic niche signals present along various points of the dorsal-ventral/anterior-posterior axis of the neural tube during development (107, 207, 208). Choosing between region-specific or whole brain organoids as a cellular model depends on the tropism of the virus under investigation. For example, if the viral tropism in the CNS is unknown, a heterogeneous model may be more suitable than a region-specific organoid. This makes stochastic CNS organoids particularly useful to study the tropism of emerging viruses. At present, PSC-derived organoid models have been used to study the infection of several neurotropic viruses, including ZIKV, HIV, HSV, SARS-CoV-2, and human cytomegalovirus (HCMV; herpesvirus) (Table 1).

Several terms to describe 3D cultures have been used in the field, sometimes interchangeably, such as spheroids, aggregates, and organoids. However, there are generally methodological distinctions that can impact how these various models may be applied to interrogate the structure and function of cellular networks in response to viral infections. Neuronal spheroids or aggregates are often not the result of the emergent, self-organizing properties of differentiating iPSCs or ESCs, but instead from assembling already differentiated cells or dedicated neural progenitors into a 3D structure (Figure 1). There can be advantages to this approach in that defined ratios of particular cell types can be co-cultured, similar to 2D cultures, but in a way that is conducive to the formation of cultures in 3D. This could allow for the development of some 3D organization, but may not recapitulate all of the cytoarchitecture that would emerge when cells undergo differentiation within the 3D structure and the cell-intrinsic processes that guide migration. Organoids that are derived directly from PSCs and embryoid bodies, as referred to here, rely more on the self-organization of differentiating cells and may result in more complex and/or physiologically-relevant architecture. However, organoids are subject to variability as well as the limitation that extensive patterning of differentiation to achieve brain-region specificity may preclude the appearance of cells from other lineages. Nonetheless, cerebral organoids are capable of generating numerous types of neural cells such as NSCs, NPCs, astrocytes, inhibitory neurons (GABAergic), excitatory neurons (e.g., glutamatergic, dopaminergic), and oligodendrocytes (209–211).

The cellular heterogeneity and brain-region specificity of organoids can reveal unexpected targets of neurotropic viruses. For example, epithelial cells were recently identified as targets of SARS-CoV-2 infection in organoid models (49, 203). In one of these studies, the susceptibility of multiple brain region-specific organoids (cortical, hippocampal, hypothalamic, midbrain) to SARS-CoV-2 infection was tested to survey the potential susceptibility of different neural cell types (49). Using this screening method, the group was able to observe that organoids with choroid plexus-like regions were the most infected by SARS-CoV-2, the pathogen responsible for the recent COVID-19 pandemic and known primarily for its disruption of the respiratory tract. Following this initial indication of CNS tropism, a protocol was developed to generate choroid plexus-specific organoids, which led to higher rates of infection in the choroid plexus-like epithelial cells than adjacent cell types. Similarly, infected choroid plexus organoids that produce cerebral spinal fluid (CSF) were infected with SARS-CoV-2, which led to damaged epithelium and a disruption of the CSF barrier in this model (50). In a study of postmortem brain samples from COVID-19 patients and age-matched controls, RNA-sequencing data revealed an upregulation of inflammatory genes and the antiviral defense gene *IFITM3* in choroid plexus cell types, although SARS-CoV-2-specific RNA was not detected in any cell type (212). While evidence of viral infection was not observed in this study, the inflammatory signals detected are consistent with the choroid plexus as a site of SARS-CoV-2-related pathology. Other studies of postmortem tissue and functioning murine systems have validated some evidence of viral RNA in brain tissue and epithelial cells (51, 213) and this remains an area of active investigation. Interestingly, a recent study suggested that dopaminergic neurons were susceptible to SARS-CoV-2 pseudo-entry virus infection, implicating a potential pathogenic locus related to neurological symptoms observed in COVID-19 patients (52). Together, these studies highlight how brain organoid models can be applied to study the tropism of new emerging viruses and suggest targets for histological investigations of postmortem and animal tissue.

### 3D models of microcephaly

A striking feature of many cortical organoid models is the presence of neural rosette-like structures that resemble some aspects of neural tube patterning of dorsal forebrain regions. These rosette-like structures contain NSCs and NPCs that give rise to postmitotic neurons that migrate radially and form distinct neuronal layers (211). In particular, cortical organoids form distinct proliferative zones, including the ventricular zone, inner subventricular zone, outer subventricular zone, cortical plate, and marginal zone, which resemble that of the developing embryonic human cortex (75, 214). A primarily human-specific outer radial glia cell layer, which is considered a hallmark of human embryonic cortex formation, has also been reported in cortical forebrain organoids (75, 76, 214). Unlike 2D monolayers that can be treated with antimitotic agents to selectively eliminate progenitors and synchronize maturation, organoids typically retain NSCs and NPCs, resulting in the continued presence of immature neurons. Some groups have focused on this aspect of cortical organoid models to study viruses that appear to target developing neural systems, such as JEV (40) and ZIKV (75).

Forebrain organoids have been particularly useful in studying the potential of neurotropic viral infections to cause structural impairments in early brain development such as microcephaly. During the ZIKV outbreak in 2015–2016, there was an increase in the birth prevalence of microcephaly that was associated with a dramatic increase in ZIKV transmission in some geographical regions (215), but whether there was a causal link was not known. To investigate if ZIKV infection could lead to structural deficits in human iPSC-derived organoids, forebrain organoids were infected with ZIKV, resulting in an overall decrease in the size of organoids and neuronal layers that resembled features of microcephaly (75). Deficits in the cytoarchitecture of the organoids and decreased size of ZIKV-infected organoids due to NPC depletion have been observed by several groups (65, 77, 78), supporting and extending the initial findings that ZIKV targets human NPCs as observed in 2D cultures (74).

Microcephaly has been implicated in other neurotropic virus infections, including HCMV. Although most instances of congenital HCMV infection are asymptomatic, symptomatic cases convey several neurological outcomes, such as primary microcephaly (216). In a recent study, HCMV-infected iPSCs were used to generate forebrain organoids and the infection impacted the formation of cortical structures in the organoids, with a minimal impact on overall growth (14). This was attributed to the focal regions of antigen detection in the organoids developed from infected iPSC lines, which mirrors clinical observations involving a wide spectrum of tissue necrosis and structural damage (217, 218). HCMV-infected organoids also presented large vacuoles and necrosis, in addition to impaired expression of the neuronal marker β-tubulin III. Another recent study (15) found that forebrain organoids infected with the HCMV strain TB40/E impaired the development and growth of cortical structures, which resulted in a significant decrease in organoid size, as well as reduced neuronal activity. One of the key differences between these studies is that infection of the iPSC line before differentiation resulted in sporadic signs of infection at later stages, whereas infection of organoids following 45 days of differentiation resulted in high levels of infection in NPCs, which could lead to a decrease in the expansion of cortical layers and an overall reduction in the size of the organoid. Other reports have suggested differences in permissiveness to HCMV between ESCs/iPSCs and later stage NPCs (219, 220). Collectively these studies reveal how tropism may evolve over time in these heterogeneous models of early brain development and may also depend on both the cell line and strain of virus. It should be noted that the differences observed in many of the human PSC culture studies compared to congenital infection outcomes may be linked to variations in tropism, growth, antibody sensitivity, and the genomic instability of low-passage wild type (e.g., TB40/E, Toledo) and high-passage laboratory strains (e.g., AD169, Towne) of HCMV used in culture (221). Thus, investigations focused on HCMV and other viruses should be designed with these associated cell culture confounds in mind, ensuring that the strain in hand is relevant to the goals of the study and can accurately reflect congenital infection *in vivo*.

In addition to HCMV and ZIKV, HSV-1-associated microcephaly has also been modeled in brain organoids where productive infection of NPCs disrupted neuroepithelial polarity, leading to a reduction in the overall size of the organoids and impaired cytoarchitecture. Importantly, many of these structural changes were distinct from that observed in 2D cultures and revealed the impact of HSV-1 infection on cell adhesion and polarity that was unique to the 3D system (29). Another study that focused on modeling HSV-1 latency reported striking differences between 2D and 3D models, showing much less reactivation of latently infected cells in organoids than in cultured neuronal networks (30). Cell-cell and cell-ECM interactions were thought to play a role in this difference, suggesting that organoids may provide a better model of viral latency and reactivation *in vivo*. Overall, these studies illustrate the utility of brain organoids to study neurotropic viruses that can impact structural integrity and cytoarchitecture in the developing brain.

### Drug discovery using organoids

Similar to 2D cultures, organoids have been useful for evaluating the efficacy of pharmacological compounds to ameliorate viral infections or mitigate functional consequences, although typically in a lower throughput format. Most of the studies to date using neural organoids have resulted from investigations of ZIKV infection, which is believed to contribute to microcephaly. One of the earliest studies used a combined approach and performed a large initial screen of more than 1,000 FDA-approved compounds in 2D cultures of iPSC-derived NPCs, followed by a smaller-scale screen of the top candidates in brain organoids to identify the most effective drugs to inhibit ZIKV replication and pathology in a 3D environment (79). Two of the target compounds, amodiaquine dihydrochloride dihydrate and hippeastrine hydrobromide, were shown to be effective in suppressing ZIKV infection in NPCs and organoids, although amodiaquine dihydrochloride dihydrate was determined to be cytotoxic at higher concentrations and only hippeastrine hydrobromide was shown to have a therapeutic effect in an *in vivo* model of infected mice. In one study focused on ZIKV, forebrain organoids were infected with the Puerto Rican ZIKV strain PRVABC 59 and a variety of antibiotics and compounds with predicted antiviral properties were tested (76). Several factors were identified that significantly reduced ZIKV mRNA in forebrain organoids including 25-hydroxycholesterol, a natural defense protein. However, some toxicity of this compound was observed, offsetting any potential rescue of cell death. Among the antibiotics tested that had previously shown some efficacy in mediating flavivirus infections, both ivermectin and duramycin dramatically reduced ZIKV mRNA while azithromycin had a minimal effect. Further, ivermectin, but not duramycin, showed some toxicity on its own. In a different study, the antibiotic enoxacin was shown to inhibit ZIKV replication and rescue cell proliferation, as well as the thinning of the ventricular zone and layered structures in organoids, in an RNAi-dependent manner (65). Although most of the targeted drug studies in these systems have focused on ZIKV, structural phenotypes have also been investigated in the context of HCMV. It was recently shown that the experimental drug maribavir partially rescued disrupted cytoarchitecture and reduced the spread of the virus, but was not sufficient to maintain calcium signaling after infection in HCMV-infected organoids (11).

Results from these studies illustrate how organoids can be used to evaluate candidate antiviral compounds to compare both toxicity and efficacy in inhibiting viral infection. Despite established protocols to target differentiation of these cultures to model specific brain regions, there is still a high degree of variability among organoids. Therefore, this 3D platform may be more useful to test targeted drugs rather than a large library of compounds. Although these early results are promising, the use of organoids as a mode of drug discovery and evaluation will likely depend upon further identification of robust and reproducible virus-induced phenotypes that can be observed in 3D culture conditions, such as dysregulated processes of cell migration or the structural integrity of certain brain regions.

### Modeling fetal brain development and drug exposure during pregnancy

Human PSC-based models recapitulate many of the processes that occur during early development and organoids in particular have been proposed as a model of neural development. In addition to cytoarchitecture and cellular diversity, brain region-specific organoids can also mirror the transcriptomic profile of human fetal brain tissue in the early stages of development. RNA-sequencing analyses of forebrain organoids have demonstrated that their transcriptome highly correlates with fetal brain tissue through the second trimester (75, 222, 223). This suggests that organoids may be a favorable model for evaluating the efficacy and safety of therapeutic drugs during pregnancy. There is currently a lack of data on the effects of many drugs on the developing fetal brain due to the limited inclusion of pregnant people in clinical trials. Much of the data available on the potential for adverse outcomes during pregnancy is obtained from observational studies following the approval of a given drug for use among the general population. There is an urgent need not only to conduct more inclusive clinical trials, but to develop additional preclinical models that could generate relevant safety information for therapeutic decision-making.

One of the clearest examples of how such information could be used to impact treatment strategies is in the case of ARVs for people living with HIV. Currently recommended regimens often require multiple drugs that rely on at least two different mechanisms of action. As the health of the mother and prevention of vertical transmission of HIV is paramount, ARVs should be taken throughout pregnancy. There are several different approved drugs within each class, but often minimal data on the impact of the drugs on neurodevelopment. A few years ago, the FDA and the European Medicines Agency issued safety notices regarding the use of the ARV drug dolutegravir. The warnings were based on the results of an initial interim observational study in Botswana suggesting that dolutegravir, if taken at the time of conception or early in pregnancy, may slightly increase the risk for severe neural tube deficits in newborns (224). Although a follow-up study suggested a statistically significant, albeit very small, increase in the risk for neural tube defects, dolutegravir is again listed as a preferred or alternative drug throughout pregnancy based on its availability, efficacy, and tolerance among most of the population (225). Using ESC-derived aggregates, one group found that dolutegravir disrupted gene expression of different developmental regulators, such as *HOXB1* and *CYP26A1*, in concentrations as low as 0.5μM (21). Further investigation of CNS-like organoids that model fetal brain development could provide a new avenue to evaluate the effects of drugs and antiviral therapies on neurodevelopment and provide novel data on the most relevant human cell types.

### Limitations of 3D cultures and emerging technologies

Organoids possess several advantages to study the effects of neurotropic viral infection and drug efficacy during pregnancy, but they are far from a perfect model. Some of the major limitations of brain region-specific organoids are the lack of vascularization, relative immaturity of constitutive cell types, and the absence of immune cells. A lack of vascularization in brain organoids leads to the deprivation of nutrients and oxygen from the media that results in the formation of a necrotic core. The necrotic core becomes more evident at later stages of culturing when the organoids grow larger in size. One strategy to overcome diffusion limits is the repeated slicing of organoids at periodic intervals to expose the inner core to factors in the media (211). This approach was shown to extend viability of the interior progenitor zones, enabling further maturation of the cortical layers and fate specification reminiscent of late-stage fetal brain development. Emerging advances in microfluidic applications may also address many of these problems. A recent study demonstrated a microfluidic device containing a brain-like ECM that facilitates nutrient and oxygen diffusion, in addition to promoting structural and functional maturation of the organoids (226). In the context of viral infection in the CNS, the absence of a BBB in brain organoids also presents a limitation to modeling physiologically-relevant processes of viral infection and drug exposure, particularly when viral infections may impact the BBB, as in the case of DENV (144). However, advances in biomedical engineering have also allowed the generation of BBB models using microfluidic devices, which presents new opportunities to study drug permeability, transport, and efficacy (227, 228).

Brain region-specific organoids typically contain immature cell populations regardless of the length of time in culture, which is why they are often used to model early development. Modeling later stages of neurodevelopment in organoids is possible, but requires extensive time in culture. A recent study showed that organoids can resemble the postnatal brain at the transcriptional level if they are cultured for more than 250 days (229). Although postmitotic neurons and some glial cell types populate organoids over time, the progenitor population is maintained as well. Unlike 2D systems in which antimitotic agents can be used to eliminate neural progenitors, this is not typically done in 3D, and the continued existence of proliferative zones may prevent full maturation of adjacent postmitotic neurons.

Another major limitation of brain organoids is the lack of immune cells, specifically microglia. Microglia are not endogenously present in cerebral organoids because they arise from a distinct lineage of the embryonic mesoderm (230). During early development, microglia derived from primitive myeloid progenitors populate and colonize the brain (231) and play important roles including surveillance of the CNS environment, synaptic and axonal pruning during development, and injury response (232). As observed in HIV, microglia have been shown to be direct targets of neurotropic viruses and can also serve as reservoirs in the adult brain (153, 233). New protocols have begun to incorporate microglia in brain organoids to account for this deficit. Recently, one study described the generation of organoids that contained innate mesoderm-derived microglia cells (234), but this approach may reduce brain-region specificity. Another recent study described a different approach to generate organoids/spheroids that contained a defined ratio of microglia cells by reaggregating NPCs with primitive macrophage progenitors (80). This method allows for the integration of cells in proportions that more closely approximate *in vivo* conditions, but the reaggregation of cells after partial differentiation precludes some of the self-directed organization and resultant cytoarchitecture that would occur through the generation of organoids that develop directly from iPSCs or ESCs. Similar to xenografts of NSC populations, transplantation of organoids to the intact circuitry of mouse models could allow for the integration of physiologically-relevant cell types to better model cell-cell interactions.

## Conclusion

Both 2D and 3D PSC-based culture strategies can provide complementary information to better understand neural pathology, neurotropism, and susceptibility to viral infections, as well as providing a platform for drug discovery (Figure 1). 2D models have the advantage of being highly scalable, easily reproducible with minimal variability, and permissive of temporal and spatial control over identified cell populations. Cell types can be defined at the molecular level (e.g., through the expression of known markers) or in terms of dynamic states (e.g., over the course of maturation). Culturing PSC-derived neural cells in monolayer cultures permits control over both of these axes of cell type identity, while 3D models offer distinct and complementary advantages. Organoids may provide data on the structural impact of various perturbagens, including viral infections. The cellular heterogeneity that occurs in organoids can be useful to identify cell types that are susceptible to emerging viruses when very little is known about the tropism within the CNS. Ultimately, the similarities to fetal brain development provide new opportunities to model *in utero* exposure to both viral infections and antiviral therapies. Although the field is continuing to evolve, current 2D and 3D cell culture models can augment existing models of viral infection. Doing so may allow for controlled investigations of human CNS cell types that can accelerate the process to determine neurotropism and associated pathology, and eventually inform the development of targeted antiviral therapeutics.

## Figures and Tables

**TABLE 1:** Representative studies using 2D and 3D human cell culture models to investigate neurotropic viruses.

Virus	2D Model	3D Model	Key findings	Methodology highlights

DENV	(8–10)	∼	Less efficient infection of neurons than ZIKV, WNV (8, 10)	Age-related effects of flavivirus infection modeled with increased passaging of iPSCs (9)
HCMV	(11–13)	(11, 14, 15)	NSC infection impairs differentiation, gene expression; infection of mature neurons induces apoptosis-associated degeneration (12) Mitochondrial dysfunction, endoplasmic reticulum stress may lead to NPC apoptosis (13) Disrupted Ca^2+^ signaling and cortical organoid structure; partial rescue with maribavir (11, 15) No effect on cortical organoid size but does compromise cytoarchitecture (14)	Different outcomes based on timing of infection in iPSCs (14) vs. organoids (15) Large populations of more homogeneous cultures allow for controlled experiments (12)
HIV	(16–20)	(20–22)	GABAergic cortical and dopaminergic neurons induce silencing and latency in infected primary human microglia (18) iPSC-derived infected microglia produce inflammatory cytokines and dysregulate EIF2 signaling across cell types (17) Pseudotyped HIV-1 infection increases degeneration of iPSC-derived spinal neurons with genetic mutation of RNA-binding protein associated with amyotrophic lateral sclerosis (19) Primary microglia integrated into cortical organoids support HIV-1 replication, alter cytokine expression (22)	Astrocyte, microglia, neuron co-culture model to study cellular cross-talk during infection and remediation (17) Microglia integrated into brain organoids, critical for the study of neuroimmune interactions (22)
HSV-1	(23–35)	(29, 30, 35–38)	HSV-1 promotes lytic changes in schizophrenia patient iPSC-derived NPCs and glutamatergic neurons (33) Identification of alternative antiviral compounds (28, 32) TLR3 and UNC93B-deficient neurons and oligodendrocytes may contribute to pathogenesis of HSE (23, 25) NPC aggregates are less susceptible to infection than monolayer NPCs (36) Latent reactivation is less efficient in brain organoids, similar to in vivo mouse models (30) Infection induces abnormal microglial activation (38)	Functional magnetic resonance imaging of HSV-1-infected schizophrenia patients (33) iPSC-based models for drug screens (28, 34) Comparison of 2D and 3D models highlights differences in phenotypes (30, 36) High-content screening in 3D cultures (37)
JEV	(39)	(40)	NPCs are highly vulnerable to JEV infection (39) JEV targets outer radial glial cells and leads to more severe infection in young ESC-derived telencephalon cortical organoids (40)	Among the few studies manipulating PSC-derived CNS cells and organoids with JEV (39, 40)
RABV	(19, 41, 42)	∼	Differences in axonal transmission between a laboratory-adapted strain and wild-type strain of rabies virus (41) RABV induces apoptosis in NPCs, but not iPSC-derived neurons or astrocytes, and suppresses interferon gamma response (42)	A novel microfluidic device to map rabies pathogenesis in human neuronal networks (41) Proteomic analysis of infected iPSC-derived neurons (42)
SARS-CoV-2	(43–48)	(47, 49–57)	Astrocytes increase infection of neurons; remdesivir inhibits infection of neurons and astrocytes (43) Neurons with the ApoE4 isoform are more susceptible to infection than ApoE3 neurons (43) Neurotropism for choroid plexus (49) ACE2 and TMPRSS2 expressed at higher levels in cerebral organoids compared to neurons and astrocytes (54)	Host-specific iPSCs shed light on susceptible human genetics (43, 44) 2D assays can inform investigation in 3D models (49)
USUV	(58)		USUV infects and induces death of iPSC-derived NSCs more efficiently than ZIKV (58)	Comparative study of flavivirus infection in iPSC-derived neural cell types (58)
VZV	(27, 59–61)	∼	First in vitro model of human iPSC-derived sensory neurons that support VZV and HSV infection (27)	Protocol to generate sensory neurons, the suspected latent reservoirs of VZV (27)
WNV	(8, 62)	∼	WNV induction of inflammatory proteins IL-8 and CCL2 (62)	Flavivirus infection in iPSCs and neural cells (8)
ZIKV	(8, 10, 19, 29, 58, 63–73)	(29, 65, 70, 72–93)	Overactivated antiviral response is detrimental in NPCs (66) Antiviral treatments effective on Vero cells did not have the same effect on iPSCs (68) Interferon gamma treatment protects NPCs from infection (10) Microglia-mediated transmission of Brazilian ZIKV to immature NPCs, increase in apoptosis (71) Exhaustion of NPCs results in cortical thinning, similar to ZIKV-associated microcephaly (70, 75, 78) Microglia in brain spheres influence inflammatory responses (83) Radial glia and early choroid plexus cells in cerebral organoids express viral entry receptor AXL (84) Sofosbuvir inhibits ZIKV infection in brain organoids (85, 86) ZIKV-NS2A disturbs formation of adherens junction and morphology of radial glia in forebrain organoids (87)	High-content screening to identify antiviral candidates (64) Miniaturized spinning bioreactor for cost-effective organoid generation (75) Automated analysis of control and infected organoid features (82)

An overview of many of the current studies using human PSCs to investigate pathology in the CNS related to viral infections and/or application of these models to identify antiviral compounds and therapeutics. A sample of key findings and methodologies are highlighted. Dengue virus (DENV); Human cytomegalovirus (HCMV); Human immunodeficiency virus (HIV); Herpes simplex virus 1 (HSV-1); Japanese encephalitis virus (JEV); Rabies virus (RABV); Usutu virus (USUV); Varicella-zoster virus (VZV); West Nile virus (WNV); Zika virus (ZIKV).
